# Novel inorganic crystal structures predicted using autonomous simulation agents

**DOI:** 10.1038/s41597-022-01438-8

**Published:** 2022-06-14

**Authors:** Weike Ye, Xiangyun Lei, Muratahan Aykol, Joseph H. Montoya

**Affiliations:** Toyota Research Institute, Energy and Materials Division, Los Altos, 94440 USA

**Keywords:** Atomistic models, Cheminformatics, Materials science

## Abstract

We report a dataset of 96640 crystal structures discovered and computed using our previously published autonomous, density functional theory (DFT) based, active-learning workflow named CAMD (Computational Autonomy for Materials Discovery). Of these, 894 are within 1 meV/atom of the convex hull and 26826 are within 200 meV/atom of the convex hull. The dataset contains DFT-optimized pymatgen crystal structure objects, DFT-computed formation energies and phase stability calculations from the convex hull. It contains a variety of spacegroups and symmetries derived from crystal prototypes derived from known experimental compounds, and was generated from active learning campaigns of various chemical systems. This dataset can be used to benchmark future active-learning or generative efforts for structure prediction, to seed new efforts of experimental crystal structure discovery, or to construct new models of structure-property relationships.

## Abstract (summary)

**Table:** 

Measurement(s)	energy above hull
Technology Type(s)	density functional theory

## Background & Summary

Crystal structure data from high-throughput density functional theory (DFT) calculations has become increasingly available, shareable, and valuable. Efforts like the Open Quantum Materials Database (OQMD)^1^, AFlowLib^2^, and Materials Project^3^ have disseminated hundreds of thousands of new crystal structures derived from both experimental reports via the Inorganic Crystal Structure Database (ICSD)^4^ and from high-throughput studies focused on specific applications and structure prototypes such as perovskites^5–7^, spinels^8,9^, garnets^10^, and Heusler alloys^11,12^.

To aid in the augmentation of these datasets, we developed a scheme to accelerate the curation of crystal structures predicted as thermodynamically stable using DFT. In our previous work, we outlined an autonomous system that, with prescriptive search input for a given chemical system, would collect new crystal structures in that chemical system using a combination of machine learning, uncertainty-estimate enabled acquisition strategies, thermodynamic phase analysis, and design-of-experiment heuristics^13^. In that system, termed Computational Autonomy for Materials Discovery (CAMD), decision-making components were encapsulated in an agent, an entity responsible for choosing new simulations based on past results.

Over the past two years, we have deployed the CAMD workflow on a scalable AWS cloud compute infrastructure which both runs the agent processes for choosing new DFT calculations from crystal structure prototypes and the associated DFT calculations themselves. In this work, we report the aggregated results of the continuous operation of the CAMD system. To date, CAMD has computed 96640 crystal structures, including 26826 within 200 meV/atom of the convex hull and 894 new ground states. The convex hull includes by default all known experimental compounds available in the OQMD at the time of the campaigns, and hence stability of new hypothetical compounds are measured against this comprehensive, realistic baseline. The dataset^14^ features a wide range of crystal structures, stabilities, and chemistries that may be used to seed experimental discovery campaigns, assist in the characterization of known materials, and enhance further active learning for crystal structure discovery.

## Methods

The CAMD workflow consists of a set of campaigns, each campaign aims to identify the stable and metastable structures (defined herein as structures with 200 meV/atom energy above the convex hull) of a specific chemical system from a pool of candidates. Put simply, a CAMD campaign is an iterative process with an research agent where, in each iteration, the agent would propose a batch of possible stable structures from the pool of candidates and send them to be validated with a DFT simulation. The simulation results are then passed back to update the agent for the next iteration, and recorded in this dataset. This process is repeated until any of the pre-set termination conditions are met. Therefore, the three most important components of the CAMD workflow are the generation of candidate crystal structures, setting of the active-learning campaigns, and the DFT calculations. The details of these components are explained in this section, and we refer the reader to our previous work for a more detailed explanation of CAMD^13^.

### Generation of candidate crystal structures

To construct this dataset, we explored 1,457 unique chemical systems with up to 4 elements. To generate the candidate crystal structures for a specific chemical system, a system of heuristic-based generation of chemical formulas followed by domain generation of structures is adapted. As the first step, the candidate stoichiometric formulas of crystals are generated by a grid-based algorithm: for chemical system *A*_*x*_*B*_*y*_*C*_*z*_..., the coefficients *x, y, z*, ... are allowed to take integer values 1, 2, 3, ... up to *g*_*max*_. Here, *g*_*max*_ is generally set to be 4 (inclusive) for binary and ternary systems. Charge balance constraints are applied to systems containing one or more of the following elements: O, Cl, F, S, N, Br, and I. This constraint is enforced based on the common oxidation states of these elements as implemented in pymatgen^15^. For these charge balanced formulas, larger values of *g*_*max*_ (up to 7) are allowed so that at least 20 candidates can be generated.

With the set of stoichiometric formulas for a chemical system, structure candidates are created using protosearch^16,17^, a crystal structure generation algorithm based on crystallographic prototypes. Starting from the ICSD entries in the OQMD database^1^ (OQMD-ICSD), 8,050 unique structural prototypes of crystals are first identified. This includes 131 unary, 1070 binary, 3196 ternary, 1970 quaternary, 1013 quinary, 542 sexinary, 104 septenary and a few higher order structures. Based on the desired compositions and the crystal prototypes, candidate crystal structures are then generated via element substitution, and unique structures are identified from the pool using the space group and Wyckoff positions. Finally, a rough optimization of the lattice constants is performed by assuming atoms are hard spheres with radii equal to 90 percent of the elements’ covalent radii, and avoiding any atomic overlap. Anisotropic scaling is also applied to relevant structures.

This process in total proposed more than 3.3 million candidate crystal structures across all the chemical systems. A set of 273 features based on composition and structure (Voronoi-based, as introduced by Ward *et al*.^18^) is calculated for each of the candidate structures using the Python package matminer^19^. These features are used in the following active learning campaign.

### Active-learning of formation energy and stability

Decision-making for each active-learning campaign is conducted by an autonomous *agent*, which in CAMD’s case includes both a machine learning model and an acquisition strategy. The model is trained and continuously updated (i.e. once every iteration) by currently available DFT data (termed the “seed data” of each iteration), and it proposes stable structures from the candidate set in each iteration by predicting and ranking the phase stabilities (i.e. energy per atom above the convex hull) of all of the remaining candidate structures in the pool. The agent simulation, benchmarking, and selection process are detailed in Ref. ^13^.

By testing various machine learning models, exploration-exploitation trade-offs (e.g. *ε*-greedy or confidence bound based methods) and uncertainty estimation techniques, an agent which uses an Adaboost regressor and a lower-confidence bound (LCB) uncertainty estimator was determined to be the most effective at discovering new materials and was therefore chosen to conduct the campaigns resulting in the included dataset. In these agents, *ε* refers to the proportion of the simulation budget devoted to randomly chosen candidates in each iteration, and the most effective agents from our benchmarking used no pure random exploration, thus have their epsilon values set to zero. Instead, the estimated uncertainty (*σ*) of the predictions from the Adaboost ensemble is used by the agent to compute a lower confidence bound (LCB) in the predicted formation energy Δ*E*_*f*_ according to $\Delta {E}_{f,LCB}=\Delta {E}_{f,AdaBoost}-\alpha \sigma$. Here *α* is a uncertainty weighting parameter and is set to be 0.5 in the chosen agent. The agent subsequently constructs a convex-hull using Δ*E*_*f,LCB*_ of candidates and the entire dataset with known *E*_*f*_, and prioritizes candidates based on their distance to the convex-hull calculated this way.

For the campaigns themselves, the research agents are initially seeded with the OQMD-ICSD dataset (34,463 structures). During each iteration of a campaign, a budget of 10 DFT calculations is allocated, where each calculation is allowed a wall-time of 8 hours on 16 CPUs on an AWS EC2 instance. Each campaign runs for at least 5 iterations, and subsequently runs until (i) the agent identifies no new materials meeting the stability criteria within any of the three most recent iterations, (ii) the campaign consumes 25% of its candidates, (iii) the campaign completes 22 iterations, or (iv) the agent predicts no new structures meet the LCB stability criteria.

### DFT parameters

All DFT calculations were performed using the Perdew-Burke-Ernzerhof (PBE)^20^ density functional with projector augmented wave (PAW)^21^ pseudopotentials as implemented in the Vienna Ab initio Simulation Package (VASP)^22^. The workflow of DFT calculations consists of a structural optimization followed by a static calculation, for which input parameters are generated using qmpy^23^ to keep consistency with the seed data derived from the OQMD. The *Experiment* API of the CAMD package submits, monitors, and fetches the output of DFT simulations to provide energy-structure pairs back into the seed data set. DFT simulations were performed in containerized environments using the AWS Batch service.

## Data Records

The data is disseminated as a json file containing key-value pairs describing each CAMD-discovered crystal structure and its associated properties. The crystal structure itself is represented in the ‘structure’ field as a pymatgen Structure object cast to a dictionary using pymatgen’s Structure.as_dict method. The primary targets for prediction ‘delta_e’ corresponding to the formation energy per atom, and the ‘stability’ corresponding to the energy above the convex hull are also included. Additional key-value pairs that may aid with sorting or filtering (e.g. ‘reduced_formula’) or machine learning (e.g. ‘features’) are detailed in Table 1. Two json files comprising the entire dataset, one with and one without computed matminer features, may be downloaded at 10.6084/m9.figshare.19601956.v1^14^.

**Table 1:** Metadata of data records.

Field	Content
data_id	A unique ID of the structure, a string combines the data_source and an integer index with an underscore.
structure	Structure file, a pymatgen Structure object
space_group	Space group symbol
chemsys	Chemical system
reduced_formula	Reduced formula
delta_e	Formation energy (eV/atom)
stability	Energy above the hull (eV/atom)
data_source	Source of the data, camd or oqmd
external_duplicate	MP or COD id corresponding to matching structures in respective databases
features	A vector of 273 features generated by a composition- and
	structure-derived material featurization method introduced by Ward *et al*.^18^, implemented in matminer^19^.

## Technical Validation

**Fig. 1:**
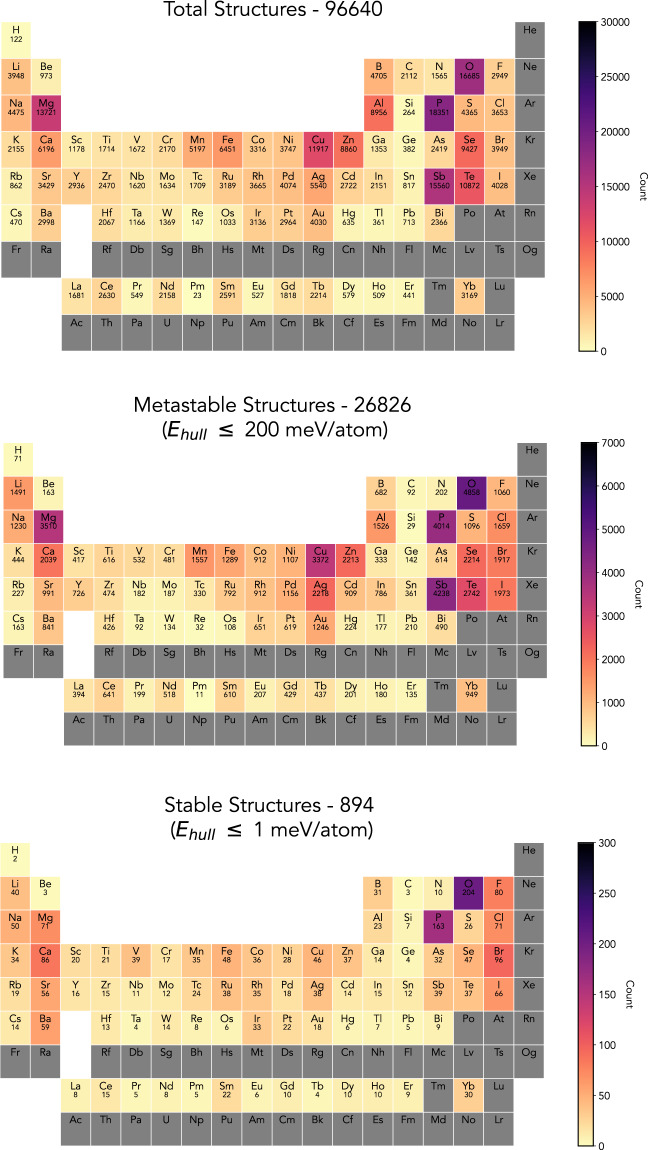
Periodic tables of CAMD discoveries: the number of structures containing a given element are labelled for the entire dataset (top), the dataset filtered to include metastable structures with E_*hull*_ ≤ 200 meV/atom, and the dataset filtered to contain only stable structures with E_*hull*_ ≤ 1 meV/atom. Elements not included in any CAMD campaign are indicated in dark gray.

There are in total of 96640 structures discovered in the aggregated CAMD campaigns, of which 26826 are within 200 meV/atom to the convex hull (metastable), and 894 are within 1 meV/atom of the convex hull. The total discovered materials cover 76 elements, with a heavy population of oxides, chalcogens (e.g., S, Se), pnictogens (e.g., P, Sb), earth alkali metals (e.g., Mg), and transition metals (e.g., Cu, Zn). The metastable structures share a similar distribution as the total discovered structures. Meanwhile, among the newly discovered stable structures, phosphides and oxides are significantly populated (Fig. 1). Considering phosphides are relatively under-explored compared to oxides in the seed data, it is promising to see that the variance in the completeness of phase information has limited influence on the CAMD agent’s ability to discover new phases.

**Fig. 2:**
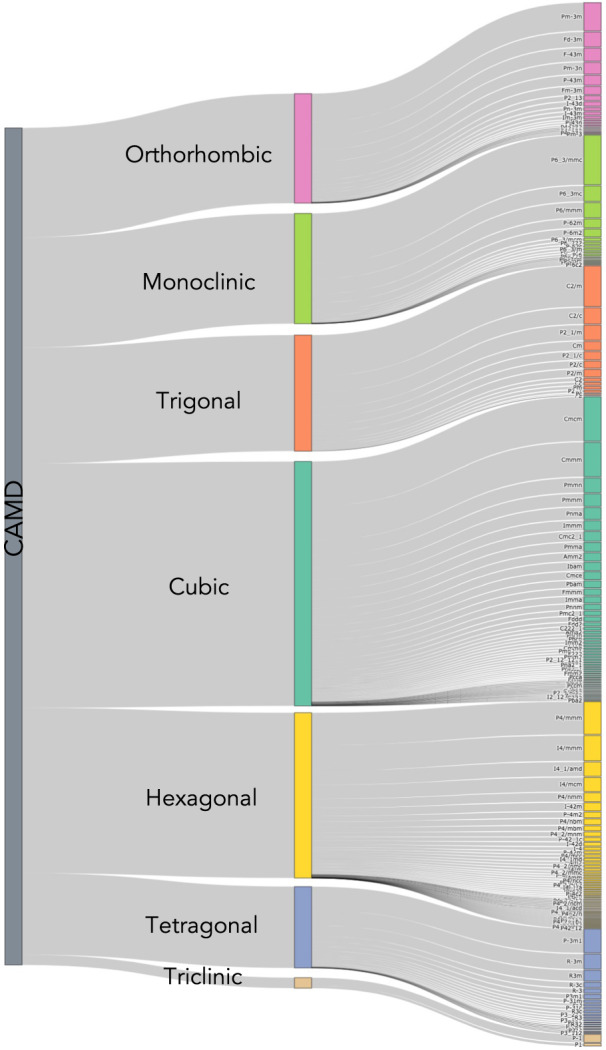
Crystal symmetries and spacegroups discovered by CAMD: the dataset is subdivided first into crystal system, e.g. cubic, and then into spacegroups. CAMD’s generative methods use crystal structure prototyping to allow for a wide range of crystal symmetries.

The discovered structures represent seven crystal systems and 181 distinct space groups, demonstrating a wide range of crystal symmetry (Fig. 2). The distribution of crystal symmetry is largely determined by the structure prototypes distilled from the OQMD-ICSD seed data. The loose positive correlation between the frequency and the symmetry of the crystal systems is expected, given that symmetry often confers stability to a crystal structure. The CAMD dataset is also largely distinct from crystal structures present in other databases, but there are duplicates we identified using pymatgen’s StructureMatcher algorithm from both the crystallography open database (COD)^24^ and the Materials Project (MP)^3^, which includes a more recent dataset from the ICSD than the version of the OQMD used for CAMD’s seed data. More specifically, 22 structures which match COD entries and 314 from the Materials Project are included in the CAMD dataset (but are excluded from the numbers and figures reported in this manuscript) and are indicated by COD or MP id, respectively, in the data records. These duplicates include a number of experimentally realized crystal structures, including a Ca_3_WO_6_ perovskite^25^, a half-heusler MgAgSb thermoelectric^26^, and an orthorhombic Na_2_NiO_2_ demonstrated as a cathode additive^27^.

**Fig. 3:**
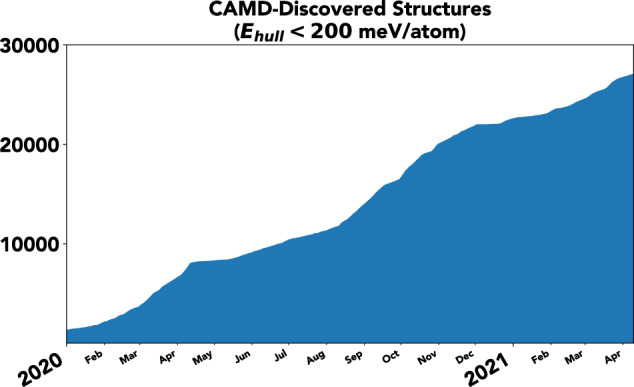
Cumulative CAMD-discovered crystal structures since 2020. Structures included have a hull energy, i.e. phase stability, below 200 meV/atom.

**Fig. 4:**
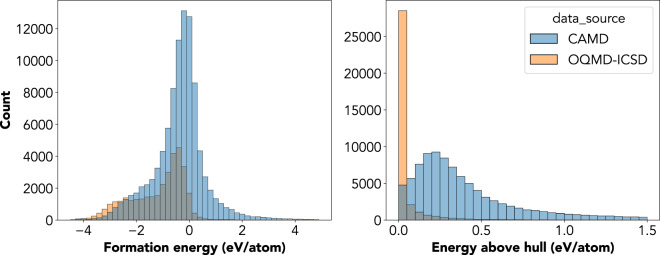
The distribution of formation energies and energy above the hull (phase stability) is shown alongside the same distribution from the ICSD as disseminated by the OQMD.

The effectiveness of the CAMD workflow is evidenced in how the dataset has grown over the past two years. To illustrate this, Fig. 3 plots the cumulative number of discovered stable and metastable structures over time. Our discovery rate is roughly linear, demonstrating that CAMD can ensure consistent discovery of new structures. In addition, the distribution of phase stabilities acquired by CAMD demonstrates how our statistical approach can ensure consistency in acquiring materials fulfilling this figure of merit. Shown in Fig. 4 are both the distributions of formation energies and energies above hull of the CAMD dataset, compared to those of the OQMD-ICSD dataset. The ICSD is naturally highly biased towards stable structures. The CAMD dataset, in contrast, seemingly peaks and decays smoothly past the cutoff stability threshold of 200 meV/atom above the hull, reflecting how CAMD includes structures with estimated uncertainties that bring them below this threshold. There is still considerable room for improvement of the CAMD agent, as nearly 75% of the computations are spent on unstable structures, which can occur either from inaccurate predictions of the mean formation energy from the agent’s AdaBoost model or from large uncertainties on potential candidates estimated from the AdaBoost ensemble^13^. However, the distribution of stabilities collected in the dataset reflects the intent to acquire structures with stabilities below the peak at 200 meV/atom encoded into the agent, from which we conjecture that it may be made even more effective in the future with more accurate models and uncertainty estimation.

**Fig. 5:**
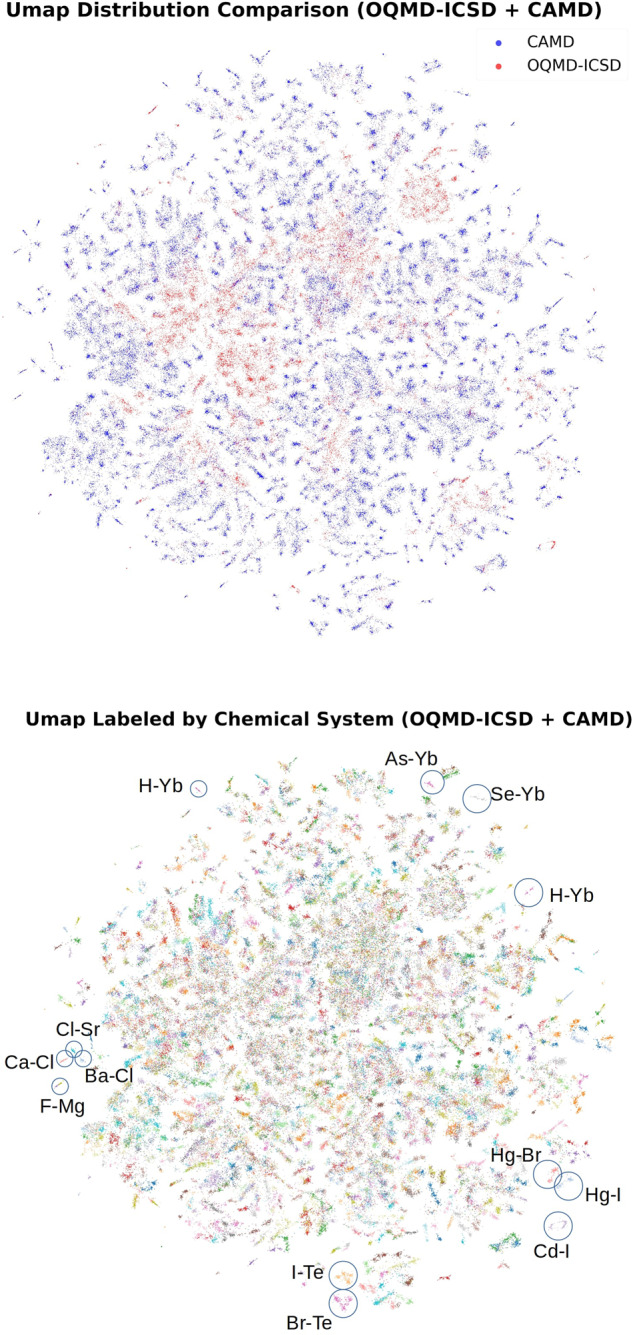
The dimensionality of the features is reduced to two by Umap (*n_neighbors* = 20*,min_dist* = 0.5) for both the ICSD and the CAMD datasets, and the distribution of the systems in both datasets is plotted. In the first figure, the scatters are colored by the data source, and in the second figure they are colored by their chemical systems. Specific chemical systems are denoted in the second figure to illustrate how clustering occurs primarily by similar chemistry.

With the diverse and strategically collected structures, the CAMD dataset is a fitting complement to the currently existing datasets and could improve modeling of prototype compounds. Figure 5 plots the distribution of the structures from the CAMD dataset compared to that from the OQMD-ICSD dataset. To generate the plots, a Umap model^28^ is trained on the combined dataset that reduces the number of features of the systems to 2 (from 274), so that they can be visualized. From the first plot, it is evident that the new CAMD dataset not only fills the gaps of the OQMD-ICSD dataset, but also significantly expands its domain. The clusters of the umap plots roughly correspond to different chemical systems, as shown in the second plot. In this plot, the scatter points are colored by the chemical systems that the crystal structures belong to. The clusters are relatively homogeneous and correspond to one specific chemical system, and structures of the same chemical system tend to cluster together. For example, looking at the *Cd-I* cluster located on the left side of the plot, it contains structures from both the CAMD and OQMD-ICSD datasets. Clusters of similar chemical systems locate near each other on the plot.

**Fig. 6:**
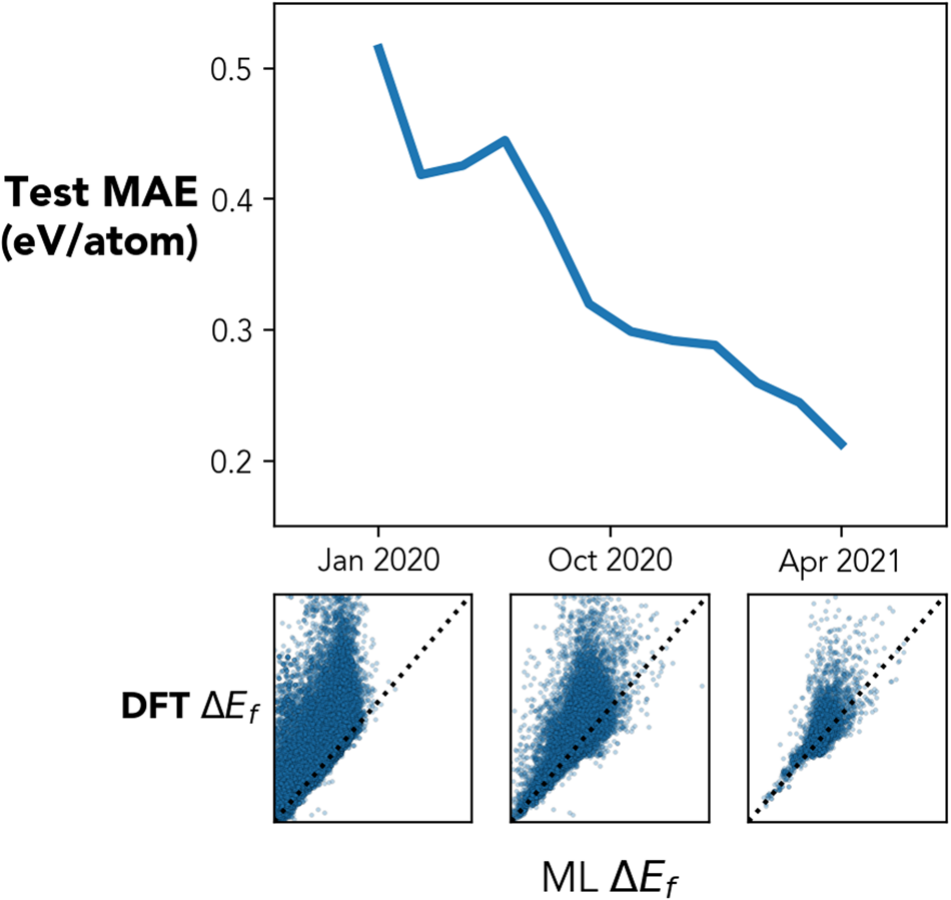
The same machine learning model, using the same composition and structural features and Adaboost model as the CAMD agent, is trained to the formation energies of materials using the CAMD dataset collected up to certain point in time. The test set is the remainder of the CAMD dataset at that point in time. The model MAE over time is plotted in the main panel on the top, showing the model is systematically improved with the growing dataset. On the bottom are the parity plots of the model predictions for the remaining CAMD test set at January 2020, October 2020 and April 2021.

Consequently, better machine learning (ML) models can be trained to predict material properties. As an example, ML models are trained to predict the formation energies of materials using the CAMD dataset collected up to different point in time and tested on the remaining dataset. The results are shown in Fig. 6, and it shows clearly that the overall accuracy of the Adaboost model used in CAMD’s agent improves systematically over time. Since the campaigns for different chemical systems are submitted sequentially, the dataset split here is different than random split of the overall CAMD dataset. On the contrary, the test set of a model - containing structures of chemical systems that were explored after the given time - is effectively a set of unseen and novel materials. At present, CAMD does not use information gained in one campaign (i.e. chemical system) in another, but this benchmark model improvement suggests that future active learning systems could benefit from a more global awareness of past acquired structures.

## Usage Notes

Sample Jupyter notebooks for analyzing the dataset and generating the figures included in this manuscript can be found at 10.6084/m9.figshare.19601956.v1^14^.

## Data Availability

The CAMD code used to generate the data described herein is available at http://github.com/TRI-AMDD/CAMD. Scripts used to generate and analyze the dataset, as well as reproduce the figures in this manuscript are all included in the above data repository.
